# Biogenic Silver Nanoparticles/Mg-Al Layered Double Hydroxides with Peroxidase-like Activity for Mercury Detection and Antibacterial Activity

**DOI:** 10.3390/molecules28155754

**Published:** 2023-07-30

**Authors:** Masira I. Chamanmalik, Arnet Maria Antony, C. V. Yelamaggad, Shivaputra A. Patil, Siddappa A. Patil

**Affiliations:** 1 Centre for Nano and Material Sciences, Jain Global Campus, Jain (Deemed-to-be University), Kanakapura, Bangalore 562112, India; masira.malik@jainuniversity.ac.in (M.I.C.); a.maria@jainuniversity.ac.in (A.M.A.); 2 Centre for Nano and Soft Matter Sciences, Survey No. 7, Shivanapura, Bangalore 562162, India; yelamaggad@cens.res.in; 3 Pharmaceutical Sciences Department, College of Pharmacy, Rosalind Franklin University of Medicine and Science, 3333 Green Bay Road, North Chicago, IL 60064, USA

**Keywords:** *Tectona grandis*, silver nanoparticles, Mg-Al layered double hydroxides, peroxidase-like activity, Hg^2+^ detection, antibacterial activity

## Abstract

Over the past decade, the attention of researchers has been drawn to materials with enzyme-like properties to substitute natural enzymes. The ability of nanomaterials to mimic enzymes makes them excellent enzyme mimics; nevertheless, there is a wide berth for improving their activity and providing a platform to heighten their potential. Herein, we report a green and facile route for *Tectona grandis* leaves extract-assisted synthesis of silver nanoparticles (Ag NPs) decorated on Mg-Al layered double hydroxides (Mg-Al-OH@TGLE-AgNPs) as a nanocatalyst. The Mg-Al-OH@TGLE-AgNPs nanocatalyst was well characterized, and the average crystallite size of the Ag NPs was found to be 7.92 nm. The peroxidase-like activity in the oxidation of *o*-phenylenediamine in the presence of H_2_O_2_ was found to be an intrinsic property of the Mg-Al-OH@TGLE-AgNPs nanocatalyst. In addition, the use of the Mg-Al-OH@TGLE-AgNPs nanocatalyst was extended towards the quantification of Hg^2+^ ions which showed a wide linearity in the concentration range of 80–400 μM with a limit of detection of 0.2 nM. Additionally, the synergistic medicinal property of Ag NPs and the phytochemicals present in the *Tectona grandis* leaves extract demonstrated notable antibacterial activity for the Mg-Al-OH@TGLE-AgNPs nanocatalyst against Gram-negative *Escherichia coli* and Gram-positive *Bacillus cereus*.

## 1. Introduction

The astonishing discrepancy between the activity of bulk materials and that of their nano-scale counterparts has served as fuel for the growth of nanocatalysis [1,2]. Since then, extensive research on nanoparticles (NPs) has been conducted and has achieved great demand in this field of research. The NPs are an excellent replacement for traditional bulk materials because of the high surface-to-volume ratio, which drastically increases their catalytic efficiency [3,4]. Thus, scientific endeavors in the synthesis of metal NPs have boomed in recent times [3,5,6]. The major downside is the agglomeration of NPs during the catalytic process, decreasing the catalytic activity in subsequent cycles and making the commercialization of NPs difficult [7]. To overcome these drawbacks, a feasible strategy is using a support material as an active support for the dispersion of NPs onto their surface. Various materials like silica, bio-macromolecules, carbon material, MoS_2_, boron nitride, layered double hydroxides (LDH), etc., have been widely explored as active supports for making sustainable heterogeneous systems [8,9,10,11,12]. The LDH, also known as hydrotalcite, are substances that have layers resembling brucite-like sheets with an intercalated anionic charge [13]. In the host layer, metal ions are aligned by a general formula ${\left[M_{1-x}^{II}M_{x}^{III}{\left(OH\right)}_{2}\right]}^{x+}{\left[A^{n-}\right]}_{x/n}\cdot yH_{2}O$ in which some of the divalent metal ions are replaced by trivalent metal ions [13,14]. The readily available hydroxyl groups on the LDH layer can be functionalized, easily making LDH a suitable support material [13,15]. In the synthesis of metal NPs, the chemical protocol implements hazardous compounds, toxic solvents, reducing agents, ligands, and additives, making them unsafe [14,16,17,18]. These toxicological problems have been addressed by simple and adaptable phytochemical-mediated biological processes in the synthesis of various metal NPs [19,20]. The utilization of three-in-one properties: reducing, capping, and stabilizing of the functional groups in phytochemicals is a boon [5,21,22,23,24]. These NPs have a wide range of applications in different fields, such as organic transformations, water treatment, medicine, enzyme-like activities, and a few others [8,25].

Nanozymes are nanomaterials with an intrinsic property to mimic natural enzymes, especially peroxidase-like activity, with catalytic activity similar to natural enzymes [26,27]. In recent years, the enzyme-mimicking properties of NPs have gained considerable interest owing to their low cost of production, ease of synthesis and handling, high stability, and tunability [28]. Regardless, the enzyme-like action of these nanozymes differs significantly from that of normal enzymes, providing a platform for further development in sensing applications [29]. Ever since the discovery of the peroxidase-like activity of Fe_3_O_4_ NPs in 2007 [30], various metal oxides, metal NPs, carbon materials, etc., have been widely explored [30]. These systems oxidize the peroxidase substrate like *o*-phenylenediamine (OPD) in the presence of H_2_O_2_ to their yellow-colored oxidized product 2,3-diaminophenazine (oxOPD). As the product is colored, the colorimetric estimation of the oxidation process is achievable, making the sensing of H_2_O_2_ a friendly strategy. Likewise, nanosystems can be utilized for their peroxidase-like activity for quantifications of heavy metals, especially toxic heavy metals like mercury [31]. Mercury is one of the most well-known heavy metals distributed in soil, water, and the atmosphere. When accumulated in the environment or human body, it can cause serious health hazards [31,32]. A straightforward and quick technology proves helpful for detecting Hg^2+^ in food, pharmaceutical, clinical, industrial, and environmental samples [33,34,35]. Thus, a colorimetric sensor with the expected prospects of high sensitivity and selectivity for Hg^2+^ detection and estimation is necessary. 

Another intriguing field of research is the development of biomaterials with antibacterial characteristics to address growing resistance towards antibiotics. This study relates to the prevention of bacterial growth and the formation of colonies, and sometimes even the death of harmful microorganisms. In recent times, bacterial infections have become a vital problem as the resistance of bacteria toward antibiotics has produced major concern [4,36]. The LDHs are well-known for being suitable functional materials and act differently depending on the type and function of the intercalated or included species. As a result, their composite on proper functionalization can act as antibacterial substances [37]. Likewise, Ag NPs have shown high antibacterial properties though limited by their toxicity in high concentrations. On the contrary, dispersing the Ag NPs onto suitable material heightens their biocompatibility and lowers toxicity, making them an excellent alternative to achieve synergistic antimicrobial activity [38]. Considering the variable size and composition of the nanoparticles, the biogenically synthesized nanocatalyst makes the catalyst bond with the microbial cells making it a capable antibacterial agent.

In this work, we synthesized Ag NPs using phytochemicals extracted from *Tectona grandis* (teak) leaves and dispersed them on Mg-Al LDH support (Mg-Al-OH@TGLE-AgNPs) as a nanocatalyst in a facile three-step synthesis. A variety of tropical countries, including Nigeria, India, Myanmar, Thailand, and Indonesia, naturally encourage the growth of the hardwood teak tree for its wood [39,40,41]. Additionally, teak leaves have been historically used in food packaging for their medicinal and flavoring properties due to the array of phytochemicals like alkaloids, terpenoids, saponins, glycosides, and sterols [42]. These phytochemicals can be adapted for the synthesis of Ag NPs [42]. The inherent peroxidase-like property of the synthesized nanocatalyst was investigated for H_2_O_2_ sensing by the colorimetric estimation of the peroxidase substrate OPD. Additionally, the enzyme mimic property of the Mg-Al-OH@TGLE-AgNPs nanocatalyst was utilized for the detection of Hg^2+^ ions by monitoring the extent of inhibition. Furthermore, the Mg-Al-OH@TGLE-AgNPs nanocatalyst displayed a noticeable antibacterial activity against Gram-positive *Escherichia coli* (*E. coli*) and Gram-negative bacteria *Bacillus cereus* (*B. cereus*) when compared to the bare support material. 

## 2. Results and Discussion

### 2.1. Synthesis of Mg-Al-OH@TGLE-AgNPs Nanocatalyst

**Scheme 1:**
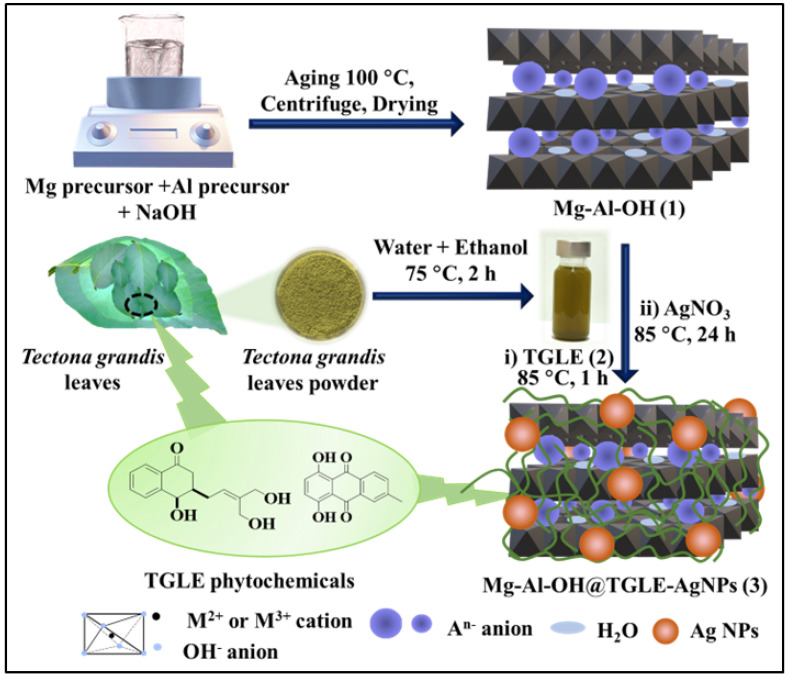
Synthesis of Mg-Al-OH-supported Ag NPs by the effective biogenic reduction in *Tectona grandis* leaf extract.

To encourage a greener approach for the synthesis of Ag NPs, we report here the biogenic synthesis of Ag NPs decorated on Mg-Al LDH support material in three superficial steps to form the Mg-Al-OH@TGLE-AgNPs nanocatalyst as demonstrated in Scheme 1. In the development of well-organized inorganic materials, several lamellar solids resembling conventional intercalation compounds have gained a lot of attention. Of them, the materials with brucite-like sheets are favored as it allows the tuning of their composition and properties [43]. The Mg-Al-OH (**1**) was prepared by a simple co-precipitation method by taking the metal precursors: Mg^2+^ and Al^3+^, in a molar ratio of 2:1 and precipitating it using the NaOH solution by controlling pH. The obtained white slurry was aged for the proper substitution of Al^3+^ in place of Mg^2+^ to form a positively charged host layer balanced by intercalated nitrate anions. The numerous hydroxyl groups on Mg-Al-OH make it an appropriate support material that is capable of functionalization in creating heterogeneous systems. The heterogeneous support also allows the uniform distribution of Ag NPs, making it catalytically available for nanozyme-like activity and heavy metal detection. On the other hand, the *Tectona grandis* leaves were chosen for their plentitude and biocompatibility to replace the detrimental and adverse chemicals required for the reduction process of metals to metal NPs. The phytochemicals in the aqueous-ethanolic extract (1:1 *v*/*v*) of the *Tectona grandis* leaves (TGLE) (**2**) act as the source of reducing and stabilizing agents in forming the Ag NPs from Ag^+^ ions. On treating the support with TGLE, the surface modification with the phytochemicals amends the Mg-Al-OH, making the immobilization of the Ag NPs possible and yielding the greyish-green color Mg-Al-OH@TGLE-AgNPs nanocatalyst (**3**). The use of phytochemicals derived from waste biomass as a three-in-one agent for reducing, capping, and stabilizing agents in the synthesis of Ag NPs makes this process green. Additionally, the overall synthesis of the nanocatalyst involves the formation of stable support material from simple precursors, including the usage of greener solvents and mild reaction conditions. Thus, the synthetic protocol of the nanocatalyst is a greener approach. 

### 2.2. Spectroscopic and Microscopic Analysis of Mg-Al-OH@TGEL-AgNPs Nanocatalyst

#### 2.2.1. GC-MS and Qualitative Analysis of TGLE

The GC-MS analysis of the ethyl acetate extract of the TGLE was carried out to investigate the presence of phytochemicals. These results displayed the presence of components like 1,2-benzenedicarboxylic acid, bis(2-methylpropyl) ester, dibutyl phthalate, 1-tricosene, phthalic acid, cyclohexyl2-pentyl ester and diamyl phthalate (Table S1). Also, the presence of saponins, alkaloids, glycosides steroids, sugar, and terpenoids was indicated by the qualitative analysis of the TGLE (Table S2, Figure S1). The action of these components leads to the fruitful reduction in Ag^+^ to Ag^0^ and the formation of a steady Mg-Al-OH@TGLE-AgNPs nanocatalyst.

#### 2.2.2. UV-Visible Analysis of TGLE

**Figure 1:**
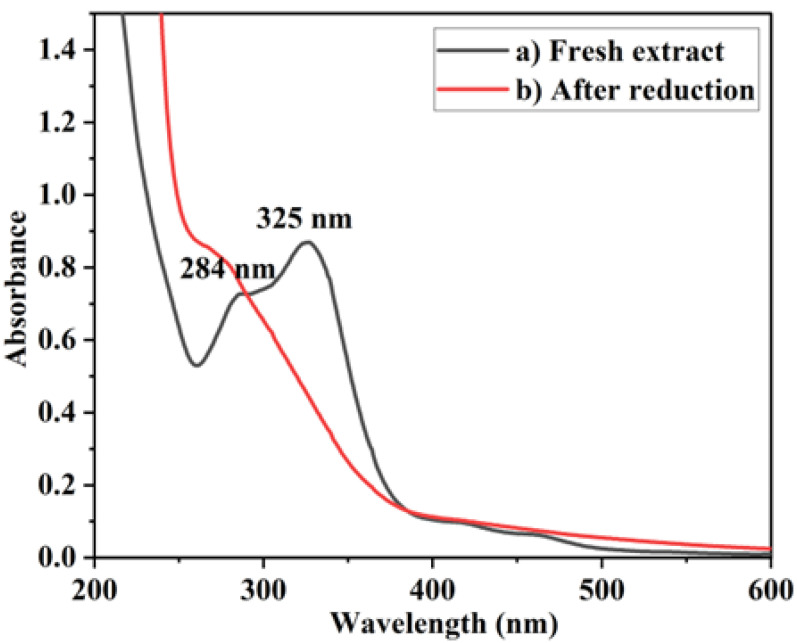
UV-Vis spectra of TGLE (a) Fresh and (b) After preparation of Mg-Al-OH@TGLE-AgNPs nanocatalyst.

The fresh and used TGLE was subjected to UV-Vis analysis to scrutinize its utility in the synthesis of the Mg-Al-OH@TGLE-AgNPs nanocatalyst. The spectrum of the fresh TGLE depicted absorbance bands at 325 and 284 nm (Figure 1, (a)), which could be indicative of the functional groups present in the phytochemicals and organic moieties ascribed to the n-π^*^ and π-π^*^ transitions. On the other hand, the characteristic bands disappeared in the spectrum of the extract obtained after being employed in the synthesis of the Mg-Al-OH@TGLE-AgNPs nanocatalyst (Figure 1, (b)), signifying the usage of phytochemicals for the reduction in Ag^+^ to Ag^0^ [22]. Additionally, the absence of any absorption peaks corresponding to Ag NPs indicates the successful formation and immobilization of Ag NPs onto the Mg-Al-OH support in the Mg-Al-OH@TGLE-AgNPs nanocatalyst.

#### 2.2.3. FT-IR Spectroscopy

**Figure 2:**
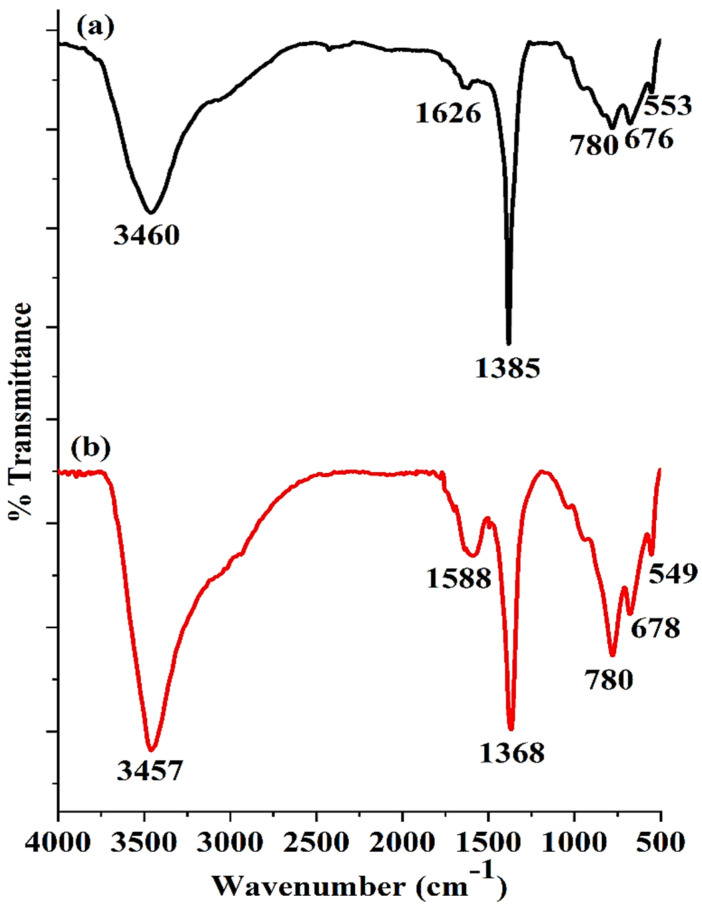
FT-IR spectra of (**a**) Mg-Al-OH and (**b**) Mg-Al-OH@TGLE-AgNPs nanocatalyst.

The formation of the Mg-Al-OH support and the Mg-Al-OH@TGLE-AgNPs nanocatalyst was established preliminarily by subjecting them to FT-IR analysis. The spectrum for Mg-Al-OH support, as depicted in Figure 1a, shows a broad absorption band at 3460 cm^−1^ of the O-H stretching vibration [14,44]. The H-O-H bending vibration of the trapped moisture and interlayer hydroxide groups peak can be seen at 1626 cm^−1^ [45], while the absorption peak at 1385 cm^−1^ represents the characteristic *v*_3_ vibration of interlayer nitrate ions [46]. The manifested peaks of Mg-O and Al-O stretching vibrations were observed at 780, 676, and 553 cm^−1^ [44,45,47]. In the synthesized Mg-Al-OH@TGLE-AgNPs nanocatalyst, the retention of the above distinctive absorption peaks was seen, although a modest shift in peak values indicates the probable functionalization of the support and interaction between the Mg-Al-OH and the Ag NPs (Figure 2b). The absorption peak at 1553 cm^−1^ could be ascribed to the unsaturated C=C (aromatic ring) and C-O (ethers, esters, and polyols) of the several phytochemicals present [48]. Thus, the successful alteration of the Mg-Al-OH surface could be proven in the formation of the Mg-Al-OH@TGLE-AgNPs nanocatalyst.

#### 2.2.4. FE-SEM Analysis

**Figure 3:**
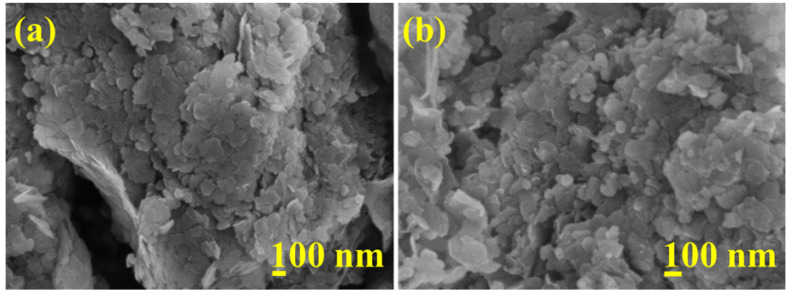
FE-SEM images of (**a**) Mg-Al-OH and (**b**) Mg-Al-OH@TGLE-AgNPs nanocatalyst.

To investigate the surface morphology of the synthesized Mg-Al-OH support and Mg-Al-OH@TGLE-AgNPs nanocatalyst, they were examined by FE-SEM analysis. This analysis demonstrates a disc or plate-like morphology for the Mg-Al-OH support (Figure 3a), which was well preserved in the developed Mg-Al-OH@TGLE-AgNPs nanocatalyst as well (Figure 3b). The slight roughening of the surface observed in the Mg-Al-OH@TGLE-AgNPs nanocatalyst could be attributed to the modification of the Mg-Al-OH surface by the phytochemicals and the formed Ag NPs.

#### 2.2.5. EDS Analysis

**Figure 4:**
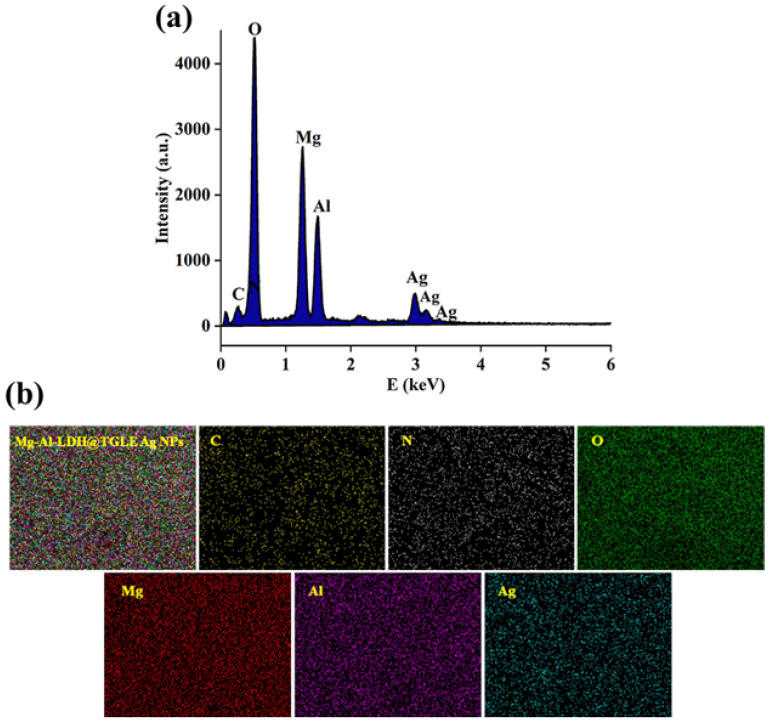
(**a**) EDS spectrum and (**b**) Elemental mapping of Mg-Al-OH@TGLE-AgNPs nanocatalyst.

The elemental composition and their distribution in the Mg-Al-OH@TGLE-AgNPs nanocatalyst were inspected by EDS analysis. The EDS spectrum revealed the characteristic signals of C, N, O, Mg, Al, and Ag (Figure 4a). Likewise, the elemental mapping of the Mg-Al-OH@TGLE-AgNPs nanocatalyst displayed the uniform distribution of these elements (Figure 4b).

#### 2.2.6. *p*-XRD Analysis

**Figure 5:**
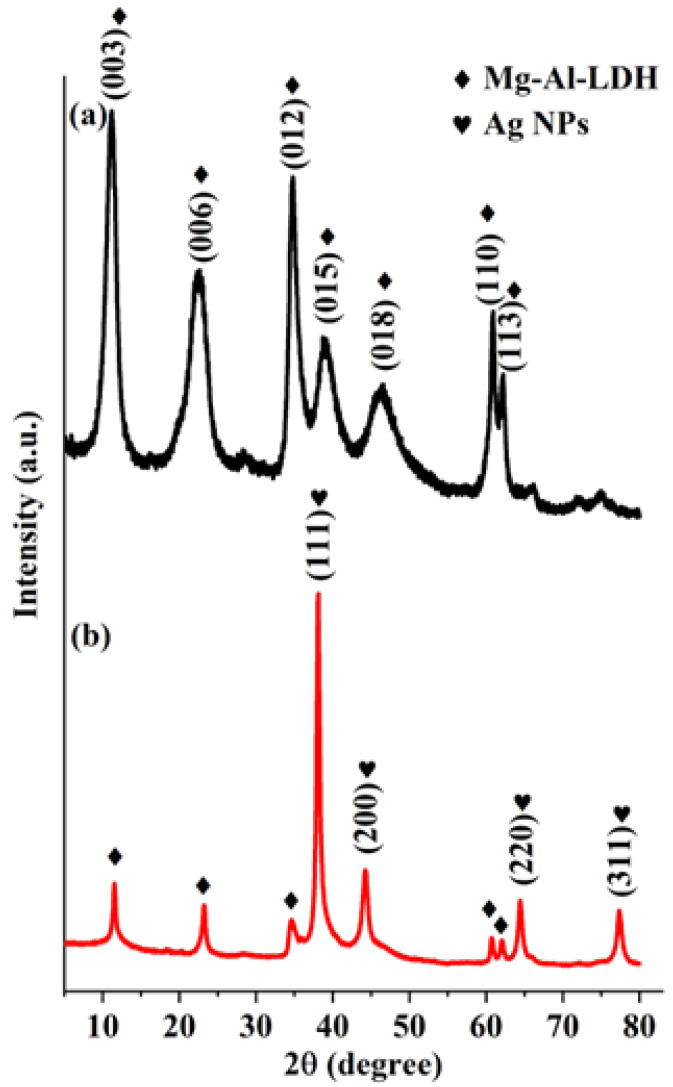
*p*-XRD pattern of (**a**) Mg-Al-OH and (**b**) Mg-Al-OH@TGLE-AgNPs nanocatalyst.

To understand the crystalline structure of the prepared Mg-Al-OH and Mg-Al-OH@TGLE-AgNPs nanocatalyst, they were subjected to *p*-XRD analysis. The characteristic diffraction peaks for the highly crystalline Mg-Al-OH corresponding to 2θ values of 11.12°, 22.46°, 35.83°, 39.02°, 46.61°, 60.85°, and 62.22° indicate the reflection planes (003), (006), (012), (015), (018), (110), and (113), as shown in Figure 5a, and are in harmony with the literature [13,49]. However, the Mg-Al-OH@TGLE-AgNPs nanocatalyst (Figure 5b), in addition to the above peaks, exhibited characteristic peaks of Ag NPs at 2θ values of 38.04°, 44.28°, 64.54°, and 77.41° for the crystalline planes (111), (200), (220), and (311) (JCPDS file no. 04-0783) [20,50,51]. Thus, we can claim that the Ag NPs were successfully formed and embedded in the Mg-Al-OH@TGLE-AgNPs nanocatalyst. From the Scherrer equation, the average crystallite size of Ag NPs was calculated to be 7.92 nm.

#### 2.2.7. Thermogravimetric Analysis

**Figure 6:**
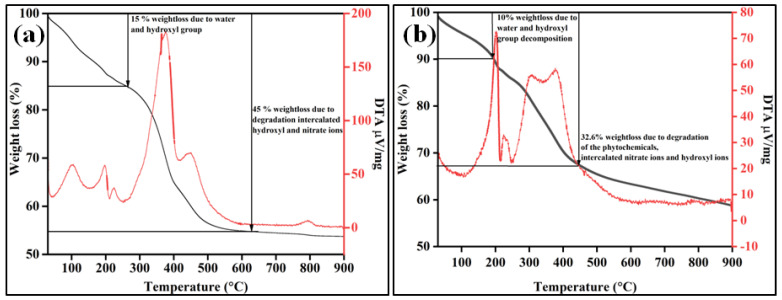
TG/DTA curves of (**a**) Mg-Al-OH and (**b**) Mg-Al-OH@TGLE-AgNPs nanocatalyst.

The TG/DTA analysis was carried out to examine the thermal stability of the synthesized Mg-Al-OH and Mg-Al-OH@TGLE-AgNPs nanocatalyst from 30 °C to 800 °C under a nitrogen environment. In Mg-Al-OH (Figure 6a), the initial 15% weight loss observed up to ~200 °C could be due to the loss of adsorbed moisture and hydroxyl groups. The following 30% could be attributed to the degradation of the intercalated nitrate ions and hydroxyl groups, leading to the formation of Mg-Al oxide. In the Mg-Al-OH@TGLE-AgNPs nanocatalyst (Figure 6b), the ~10% weight loss that was detected for up to ~150 °C might be due to the loss of moisture and hydroxyl groups, as well as the disintegration of the physisorbed phytochemicals. The broad exothermic peak around 250–450 °C indicates the decomposition of organic moieties. Thus, we can conclude that the Mg-Al-OH@TGLE-AgNPs nanocatalyst was stable up to about 250 °C.

### 2.3. Peroxidase-like Activity of Mg-Al-OH@TGLE-AgNPs Nanocatalyst

**Scheme 2:**
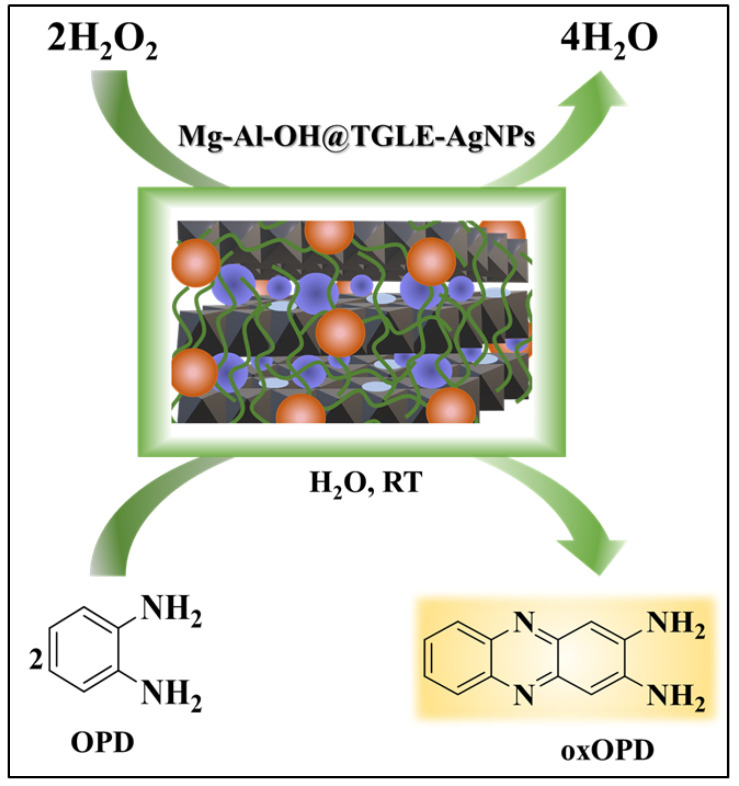
The peroxidase-like activity of Mg-Al-OH@TGLE-AgNPs nanocatalyst in the sensing of H_2_O_2_ using OPD as the peroxidase substrate.

**Figure 7:**
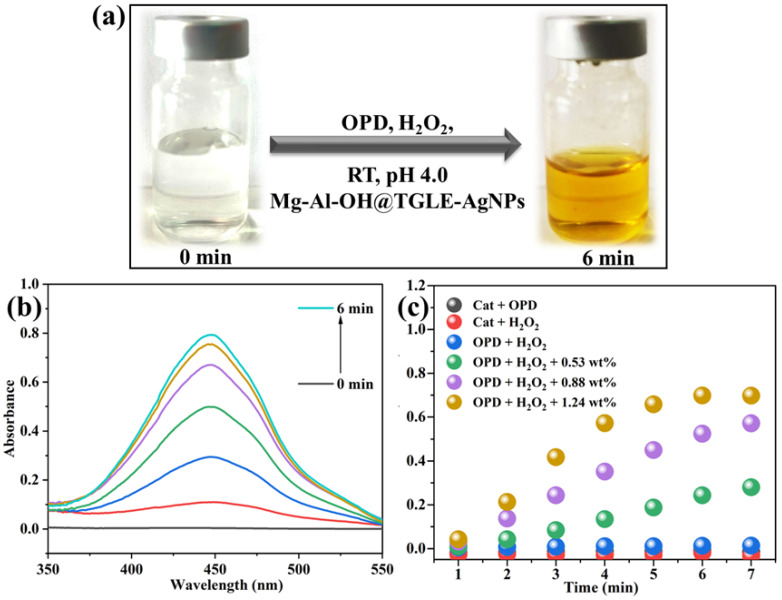
(**a**) Change in color in H_2_O_2_ sensing using Mg-Al-OH@TGLE-AgNPs nanocatalyst and OPD at time intervals of 0 and 6 min, (**b**) Absorption spectra with respect to time for the oxidation of OPD by H_2_O_2_ using Mg-Al-OH@TGLE-AgNPs nanocatalyst (1.24 wt% Ag) and (**c**) Plot of absorbance versus time at various reaction conditions.

To investigate the inherent peroxidase-like activity of the synthesized Mg-Al-OH@TGLE-AgNPs nanocatalyst, H_2_O_2_ was quantified using OPD as the peroxidase substrate in an acetate buffer of pH 4 (Scheme 2). The extent of oxidation was monitored colorimetrically as the oxidized product; oxOPD was yellow-colored with a λ_max_ of 447 nm, while both the substrates, OPD and H_2_O_2,_ were colorless (Figure 7a,b). Further, various control experiments were performed to establish the intrinsic nanozyme activity of the Mg-Al-OH@TGLE-AgNPs nanocatalyst. As expected, the results revealed no oxidation in the absence of OPD or H_2_O_2_, although a slow oxidation process occurred whenvoid of the Mg-Al-OH@TGLE-AgNPs nanocatalyst might have resulted from the formation of a minor quantity of ·OH radicals in the reaction. The availability of the Mg-Al-OH@TGLE-AgNPs nanocatalyst in the H_2_O_2_+OPD medium resulted in an abrupt increase in the rate of oxidation over a short time interval (Figure 7b). We observed a sharp increase in the slope with an increasing nanocatalyst loading (0–1.24 wt% Ag), complementing its peroxidase-like activity in OPD oxidation by H_2_O_2_.

#### 2.3.1. Effect of Parameters in the Peroxidase-like Activity of the Mg-Al-OH@TGLE-AgNPs Nanocatalyst

**Figure 8:**
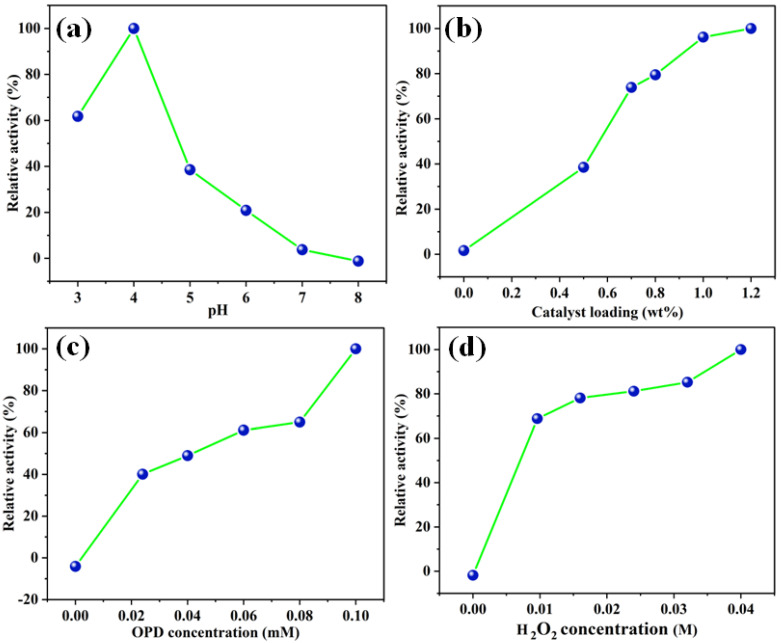
Comparison of the % of relative activity for the peroxidase-like activity of Mg-Al-OH@TGLE-AgNPs nanocatalyst with respect to (**a**) pH, (**b**) Nanocatalyst loading, concentration of (**c**) OPD and (**d**) H_2_O_2_.

Parameters such as the pH of the acetate buffer, catalyst loading, and concentrations of OPD and H_2_O_2_ were studied to examine the efficiency of the Mg-Al-OH@TGLE-AgNPs nanocatalyst toward peroxidase-like activity (Table S3). The pH was evaluated in the range of 3–8 using OPD (1 mM, 250 μL) and H_2_O_2_ (0.4 M, 250 μL) in the presence of Mg-Al-OH@TGLE-AgNPs nanocatalyst (1.24 wt% Ag) (Figure 8a). The excellent peroxidase-like activity was displayed at pH 4. In addition, moving to the alkaline range, a drastic decrease in the activity might be due to the breakdown of H_2_O_2_ in an alkaline medium. The pH-dependent study revealed that peroxidase-like activity is related not only to the substrates and nanocatalyst but to the pH as well [52]. Further, the analysis was extended to optimize the Mg-Al-OH@TGLE-AgNPs nanocatalyst loading (0–1.24 wt% Ag) for the optimal oxidation conditions, maintaining the same concentrations of OPD and H_2_O_2_ at pH 4 (Figure 8b). As expected, the increase in the nanocatalyst loading enhanced the oxidation process. In addition, the % of the relative activity with varying concentrations of the substrates OPD (0–0.1 mM) and H_2_O_2_ (0–0.04 M) are portrayed in Figure 8c,d, keeping the counter substrate concentration constant.

#### 2.3.2. Kinetic Analysis of the Peroxidase-like Activity of Mg-Al-OH@TGLE-AgNPs Nanocatalyst

The Michaelis–Menten Equation (1) and Lineweaver–Burk plot (2) were applied to determine the kinetic parameters of the enzyme-substrate affinity. The respective equations are as follows:

(1) $$V_{0}=\frac{V_{max}\left[S\right]}{K_{m}+\left[S\right]}$$

(2) $$\frac{1}{V_{0}}=\frac{K_{m}}{V_{max}\left[S\right]}+\frac{1}{V_{max}}$$

**Figure 9:**
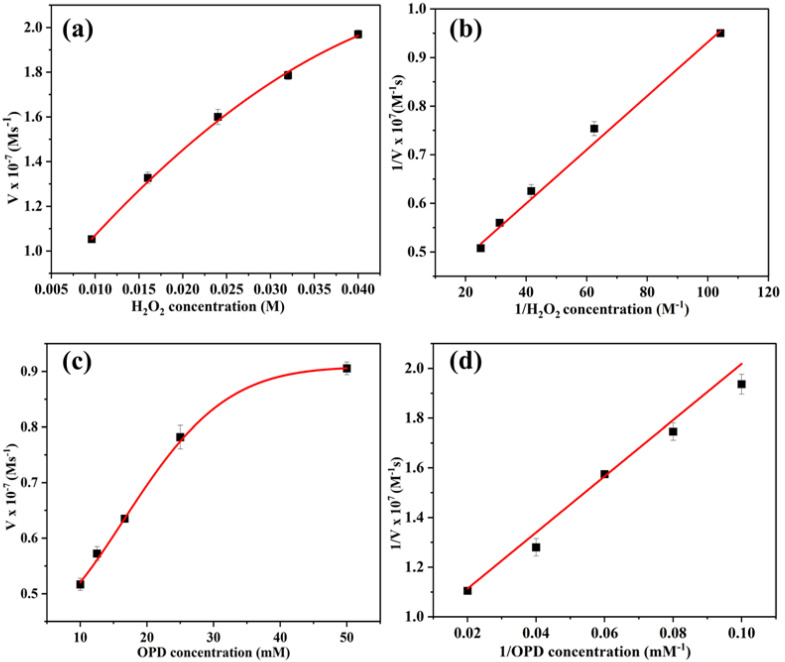
The steady-state kinetics assay and double-reciprocal plot (average of triplicate studies) for the peroxidase-like activity of the Mg-Al-OH@TGLE-AgNPs nanocatalyst for (**a**,**b**) H_2_O_2_ (with 0.1 mM OPD and 0–0.04 M H_2_O_2_) and (**c**,**d**) OPD (with 0.04 M H_2_O_2_ and 0–0.1 mM OPD).

In both equations, ‘V_0_’ is the initial rate and ‘V_max_’ is the optimum rate of conversion in terms of concentration/time, ‘S’ is the substrate concentration, and K_m_ is the Michaelis constant. By fitting the absorbance data obtained for OPD and H_2_O_2_ into the above equations, K_m_ and V_max_ were computed to be 13.03 M and 1.18 × 10^−7^ Ms^−1^ for OPD (R^2^ = 0.994), while 1.46 × 10^−2^ M and 2.66 × 10^−7^ Ms^−1^ for H_2_O_2_ (R^2^ = 0.998), respectively (Figure 9). K_m_ denotes the affinity between the substrate molecules and the Mg-Al-OH@TGLE-AgNPs nanocatalyst for the peroxidase-like activity. In addition, the limit of detection of 0.05 and 4.1 mM were obtained for OPD and H_2_O_2_, respectively.

### 2.4. Application of Mg-Al-OH@TGLE-AgNPs Nanocatalyst in Sensing of Mercury

**Scheme 3:**
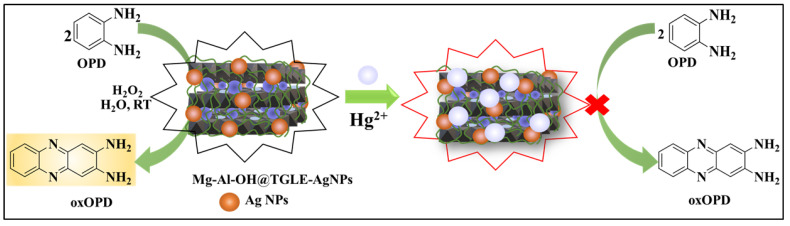
Schematic illustration of the colorimetric assay for mercury detection using Mg-Al-OH@TGLE-AgNPs nanocatalyst.

**Figure 10:**
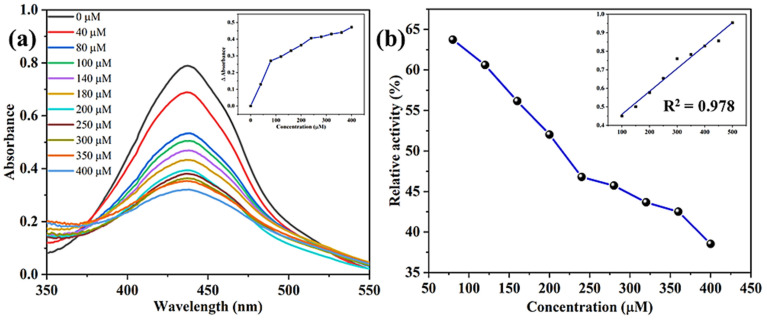
(**a**) Absorption spectra of oxOPD with varying Hg^2+^ concentration (Inset: Plot of change in absorbance versus concentration) and (**b**) % relative activity with varying Hg^2+^ concentration (Inset: linear relationship between absorbance and concentration).

To further highlight the potential application of the Mg-Al-OH@TGLE-AgNPs nanocatalyst, the quantification of the heavy metal pollutant Hg^2+^ was tested. The unique property of mercury to form an amalgam with other metals, thereby leading to the effective inhibition of the metal’s catalytic activity, could be exploited when estimating mercury (Scheme 3) [53]. Consequently, the addition of Hg^2+^ suppressed the oxOPD formation due to the Ag-amalgam formed between the Ag NPs on the Mg-Al-OH@TGLE-AgNPs nanocatalyst and Hg^2+^; this was accompanied by a decrease in the absorbance (Figure 10a). As the concentration of Hg^2+^ increased in the medium, inhibition also increased, resulting in the accomplishment of the sensitive detection of Hg^2+^ ions. In order to confirm the quenching ability, a series of experiments with different concentrations of Hg^2+^ ions (0–400 μM) were conducted. Predictably, in the absence of Hg^2+^, the peroxidase-like activity of the Mg-Al-OH@TGLE-AgNPs nanocatalyst was unaffected, while the increasing concentration of Hg^2+^ led to an almost linear decline in the absorbance (Figure 10b). The linear range for Hg^2+^ ion detection was found to be 80–400 μM with a detection limit of 0.2 nM.

### 2.5. Antibacterial Activity of Mg-Al-OH@TGLE-AgNPs Nanocatalyst

**Figure 11:**
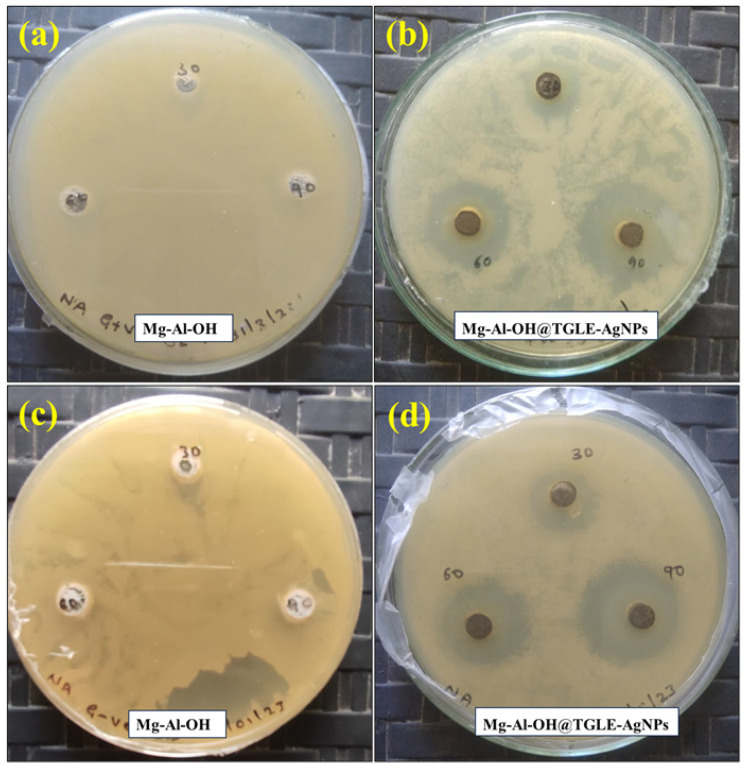
Photographs showing the antibacterial activity of (**a**,**c**) Mg-Al-OH and (**b**,**d**) Mg-Al-OH@TGLE-AgNPs nanocatalyst against (**a**,**b**) *E. coli* and (**c**,**d**) *B. cereus*.

To extend the nanocatalyst to the medicinal field, the Mg-Al-OH support and biogenically synthesized Mg-Al-OH@TGLE-AgNPs nanocatalyst were tested for their antibacterial activity. The materials were assessed by comparing the zone of inhibition (ZOI) of the pathogenic strains, Gram-negative bacteria *E. coli* and Gram-positive *B. cereus,* with the standard drug Ciprofloxacin (Figure 11). The general mechanism involves the interaction between the bacterial cell wall and the nanoparticles that leads to cell wall rupture. In the case of Gram-negative strains, the cell wall is thinner, while Gram-positive strains are thicker [36]. The presence of positive charges from the developed Mg-Al-OH@TGLE-AgNPs nanocatalyst and the negatively charged bacterial cell wall effectively interact and interrupt the cell structure and membrane permeability, ultimately leading to puncturing of the bacterial cell [54,55]. Additionally, the silver metal ions with a strong affinity for DNA and proteins readily destroy the DNA and protein activities by binding to them [36,54]. According to the ZOI, the Mg-Al-OH@TGLE-AgNPs nanocatalyst demonstrated greater antimicrobial activity than the bare support material Mg-Al-OH (Table 1). The greener approach in the synthesis and dispersion of the Ag NPs, and the presence of various phytochemicals synergistically boosted the antibacterial property without the worry of toxicity. When the Ag NPs interacted with the bacterial cell wall, rupturing could occur, interfering with cell function and ultimately causing the death of the bacteria [4].

## 3. Experimental Section

### 3.1. Materials

All solvents were used as received. The chemicals Mg(NO_3_)_2_·6H_2_O, Al(NO_3_)_2_·9H_2_O, AgNO_3_, NaOH, CH_3_COONa, OPD, H_2_O_2_, glacial acetic acid and reagents for qualitative analyses were purchased from Avra and Sigma-Aldrich chemical companies and used without further purification. The *Tectona grandis* (teak leaves) were collected from the Jain University garden, Jain Global Campus, Bangalore, Karnataka, India. All the reactions were performed in oven-dried glassware under aerobic conditions, with stirring accomplished by a magnetic stirrer unless otherwise noted. Heating was accomplished by a silicone oil bath.

### 3.2. Instrumentation and Analyses

Fourier transform infrared (FT-IR) spectra were recorded with a PerkinElmer Spectrum Two spectrometer. Field-emission scanning electron microscopy (FE-SEM) with energy-dispersive X-ray spectroscopy (EDS) to observe the morphology and determine elemental distributions, respectively, were conducted with a JEOL model JSM7100F. Powder X-ray diffraction (*p*-XRD) patterns were obtained using a BrukerAXS D8 Advance. Thermogravimetric differential thermal analysis (TG/DTA) was carried out with a PerkinElmer Diamond TG/DTA with a heating rate of 10.0 °C min^−1^ in a nitrogen atmosphere. All absorbance readings were recorded using a UV-Visible spectrophotometer (UV-1900, Shimadzu, Japan) from 200 to 800 nm. The peroxidase-like activity was carried out at room temperature under an aerobic atmosphere in aqueous medium.

### 3.3. Synthesis of Mg-Al Layered Double Hydroxides (Mg-Al-OH) *(**1**)*

The Mg-Al-OH (**1**) was synthesized by a straightforward co-precipitation method. The metal precursors Mg(NO_3_)_2_·6H_2_O (15.38 g, 0.6 M) and Al(NO_3_)_2_·9H_2_O (11.25 g, 0.3 M) were dissolved separately in distilled water (100 mL). The prepared metal precursors were taken in a 2:1 *v*/*v* ratio (Mg:Al) and precipitated using NaOH solution (0.25 M) until the pH attained was 9.5. The Mg-Al-OH suspension obtained was then aged for 24 h at 100 °C, washed with distilled water to achieve a neutral pH, followed by ethanol (2 × 20 mL), and dried at 80 °C for 12 h.

### 3.4. Preparation of Tectona Grandis Leaves Extract (TGLE) *(**2**)*

The *Tectona grandis* leaves were cleaned sequentially with tap water and distilled water before shade drying. Then, the shade-dried leaves were chopped into tiny bits and processed into powder using an electric blender. In total, 2 g of the powdered leaves were extracted with EtOH:H_2_O (200 mL, 1:1 *v*/*v*) in a conical flask with a funnel placed on the mouth of the flask at 75 °C for 2 h. The supernatant was collected after centrifugation at 3000 rpm for 5 min to remove the leaf residue and filtration. The particle-free clean extract (TGLE) (**2**) was stored in the refrigerator at 4 °C for further use.

### 3.5. Green Synthesis of Ag NPs Entrenched TGLE Dispersed on Mg-Al-OH (Mg-Al-OH@TGLE-AgNPs) *(**3**)*

To Mg-Al-OH (1 g) (**1**) taken in a round bottom flask, 160 mL of TGLE (**2**) was added. The resulting mixture was stirred for 1 h at 85 °C. Following this, an aqueous solution of AgNO_3_ (10 mM, 50 mL) was added and further stirred at the same temperature for 24 h. The Mg-Al-OH@TGLE-AgNPs (**3**) formed were separated, washed with water (2 × 25 mL) and methanol (2 × 25 mL), and dried at 80 °C for 12 h.

### 3.6. Peroxidase-like Activity of Mg-Al-OH@TGLE-AgNPs Nanocatalyst

The OPD and H_2_O_2_ solutions were prepared in distilled water. Typically, the OPD (1.0 mM, 250 μL), H_2_O_2_ (0.4 M, 250 μL), and Mg-Al-OH@TGLE-AgNPs nanocatalyst (1.24 wt% Ag) were added to acetate buffer (1.5 mL, pH 4) and taken in a quartz cell. The volume is made up of 2.5 mL of distilled water. The development of the yellow color by the oxidation reaction was monitored using a UV-Visible spectrophotometer periodically [25].

### 3.7. Mercury Detection Using Mg-Al-OH@TGLE-AgNPs Nanocatalyst

The colorimetric detection of aqueous Hg^2+^ ions was evaluated at room temperature. The Mg-Al-OH@TGLE-AgNPs nanocatalyst (1.24 wt% Ag), H_2_O_2_ (0.04 M, 250 μL), and Hg^2+^ solution (0–400 μM) were added to the acetate buffer (1.5 mL, pH 4), and incubated for 5 min. Finally, OPD (1.0 mM, 250 μL) was added and further incubated for 20 min. The absorbance of the resultant mixture was observed using a UV-Visible spectrophotometer [56,57].

### 3.8. Anti-Bacterial Activity of Mg-Al-OH@TGLE-AgNPs Nanocatalyst

The antimicrobial activity of the Mg-Al-OH and Mg-Al-OH@TGLE-AgNPs nanocatalyst has been investigated by employing the agar disc diffusion method. The *E. coli* (Gram-negative, 1 mL in 600 μL) and *B. cereus* (Gram-positive, 1 mg in 1 mL) strains as test organisms and Ciprofloxacin (30 μL) as the standard drug were selected. The test organisms were incubated overnight at 37 °C in a bacteriological incubator before spreading using an L-shaped glass spreader onto 100 mm Petri plates containing 25 mL of the nutrient agar and using the spread plate method. The test samples (30, 60, and 90 μg) and standard drug were loaded, and the Petri plates were further incubated at 37 °C for 24 h. After the incubation period, the results were evaluated and noted by measuring the diameter of the zone of inhibition, which was formed around the well.

## 4. Conclusions

In brief, a stable and green Mg-Al-OH@TGLE-AgNPs nanocatalyst was synthesized using a straightforward, environmentally friendly, and simple synthesis process. This was then characterized through microscopic and spectroscopic techniques to confirm its chemical composition, structure, surface morphology, and thermal stability. Further, the Mg-Al-OH@TGLE-AgNPs nanocatalyst showed admirable peroxidase-like activity for H_2_O_2_ sensing with the peroxidase substrate OPD colorimetrically. The Mg-Al-OH@TGLE-AgNPs nanocatalyst was then extended as a nanosensor for Hg^2+^ ion detection, where it portrayed very good competence with a linear detection range and LOD of 80–400 μM and 0.2 nM, respectively. The feasibility and selectivity of the nanocatalyst’s vast potential for on-site sensing technologies could be further investigated. Additionally, the biogenic Mg-Al-OH@TGLE-AgNPs nanocatalyst showed noticeable results for the antimicrobial activity against pathogenic bacteria, *E. coli* and *B. cereus*, adding to its versatility.

## Figures and Tables

**Table 1:** Zone of inhibition of Mg-Al-OH and Mg-Al-OH@TGLE-AgNPs nanocatalyst against *E. coli* and *B. cereus*.

Sample	Mean ZOI Observed in Case of *E. coli*			Mean ZOI Observed in Case of *B. cereus*		
**Ciprofloxacin**	27 mm			24 mm		
**Mg-Al-OH**	30 μg	60 μg	90 μg	30 μg	60 μg	90 μg
	3 mm	6 mm	8 mm	-	2 mm	4.5 mm
**Mg-Al-OH@TGLE-AgNPs**	30 μg	60 μg	90 μg	30 μg	60 μg	90 μg
	8 mm	12 mm	14 mm	8 mm	9 mm	18 mm

## Data Availability

No new data were created or analyzed in this study. Data sharing is not applicable to this article.

## Supplementary Materials

The following supporting information can be downloaded at: https://www.mdpi.com/article/10.3390/molecules28155754/s1, Figure S1. Images of the test results for the qualitative analysis of phytochemicals [42]; Table S1. GC-MS analysis of phytochemicals [58]; Table S2. Qualitative analysis of phytochemicals; Table S3. Relative activity (%) of the Mg-Al-OH@TGLE-AgNPs nanocatalyst with respect to pH, catalyst loading, OPD and H_2_O_2_ concentrations [25].

## Sample Availability

Samples of the compounds are available from the authors.
