# Supplementary material for: Biogenic Silver Nanoparticles/Mg-Al Layered Double Hydroxides with Peroxidase-like Activity for Mercury Detection and Antibacterial Activity
Source: Molecules. 2023 Jul 30;28(15):5754. doi: 10.3390/molecules28155754 (PMC10421139; doi:10.3390/molecules28155754)
Supplement: Supplementary file 1 [file molecules-28-05754-s001.zip › molecules-2509044-supplementary.pdf]

Supporting Information

Biogenic Silver Nanoparticles/Mg-Al Layered Double Hydroxides with Peroxidase-like Activity for Mercury Detection and Antibacterial Activity

Masira I. Chamanmalik [1], Arnet Maria Antony [1], C. V. Yelamaggad [2], Shivaputra A. Patil [3,*] and Siddappa A. Patil [1,*]

[1] Centre for Nano and Material Sciences, Jain Global Campus, Jain (Deemed-to-be University), Kanakapura, Bangalore 562112, India
[2] Centre for Nano and Soft Matter Sciences, Survey No. 7, Shivanapura, Bangalore 562162, India
[3] Pharmaceutical Sciences Department, College of Pharmacy, Rosalind Franklin University of Medicine and Science, 3333 Green Bay Road, North Chicago, IL 60064, USA
* Correspondence: shivaputra.patil@rosalindfranklin.edu (S.A.P.); p.siddappa@jainuniversity.ac.in or patilsiddappa@gmail.com (S.A.P.)

Table of contents

1. GC-MS analysis of phytochemicals -- 3
2. Qualitative analysis of phytochemicals -- 4
3. Images of the test results of qualitative analysis of phytochemicals ------------------------------- 5
4. Relative activity (%) of the Mg-Al-OH@TGLE-AgNPs nanocatalyst with respect to pH, catalyst loading, OPD and H_2O_2 concentrations --- 5
5. References --- 6

Table S1. GC-MS analysis of phytochemicals.

Sl. No.	RT (min)	Area (%)	Compound
1.	8.912	8.08	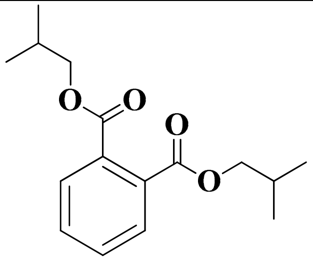 Diisobutyl phthalate
2.	8.857	23.56	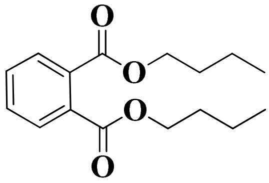 Dibutyl phthalate
3.	8.431	4.46	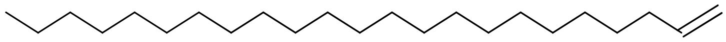 1-Tricosene
4.	9.132	4.85	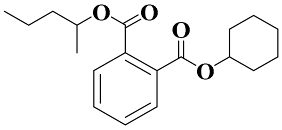 Cyclohexyl pentan-2-yl phthalate
5.	9.180	5.36	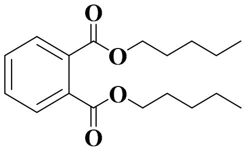 Dipentyl phthalate

Table S2. Qualitative analysis of phytochemicals [2].

Sl. No.	Test	Observation	Inference
1.	**Test for saponins**		
	Plant extract (aq.) shaken with water in the test tube	Development of stable foam	Saponins are present
2.	**Test for alkaloids**		
	Plant extract + few drops of Mayer's reagent	Formation of precipitate	Alkaloids are present
3.	**Test for terpenoids**		
	Plant extract + few Drops of conc. H_2SO_4	Formation of the pale yellow layer down	Terpenoids are present
4.	**Test for glycosides and sterols**		
	Plant extract + 2 mL chloroform + Conc. H_2SO_4	Reddish brown interface formation	Glycosides and sterols are present
5.	**Test for sugars**		
	Plant extract + 2ml distilled water + Molish's reagent	Purple violet ring in interference	Sugars are present

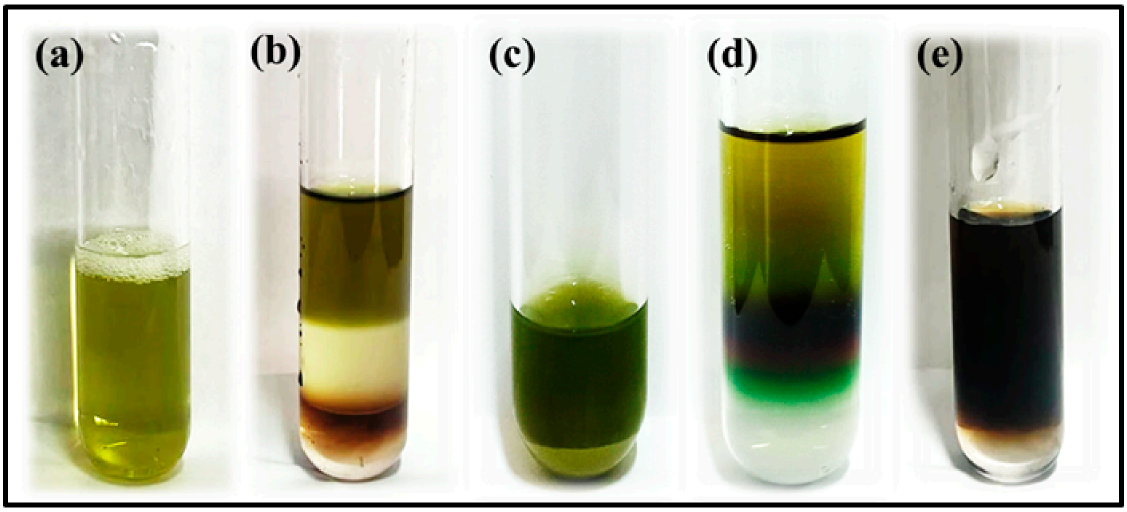

Figure S1. Images of the test results of qualitative analysis of phytochemicals [1].

Table S3. Relative activity (%) of the Mg-Al-OH@TGLE-AgNPs nanocatalyst with respect to pH, catalyst loading, OPD and H_2O_2 concentrations [3].

**pH**	3	4	5	6	7	8
**Relative activity (%)**	61.738	100	38.525	20.863	3.725	1.238
**Catalyst loading (wt% Ag)**	0	0.53	0.71	0.88	1.06	1.24
**Relative activity (%)**	1.638	38.488	73.863	79.475	96.175	100
**OPD concentration (mM)**	0	0.024	0.04	0.06	0.08	0.1
**Relative activity (%)**	4.075	40.063	48.925	61.063	64.938	100
**H_2O_2 concentration (M)**	0	0.009	0.016	0.024	0.032	0.040
**Relative activity (%)**	1.825	68.788	78.113	81.188	85.200	100

References

1. Vyas, P.; Yadav, D. K.; Khandelwal, P., Tectona grandis (teak)–A review on its phytochemical and therapeutic potential. *Nat. Prod. Res.* **2019,** 33, (16), 2338-2354.

2. Khatri, P.; Rana, J.; Jamdagni, P.; Sindhu, A., Phytochemical screening, GC-MS and FT-IR analysis of methanolic extract leaves of Elettaria cardamomum. *Int. J. Res.* **2017,** 5, (2), 213-224.

3. Antony, A. M.; Yelamaggad, C.; Patil, S. A., Palladium nanoparticles decorated on functionalized graphitic carbon nitride as an efficient and retrievable nanocatalyst for organic dye degradation and hydrogen peroxide sensing. *Mater. Chem. Phys.* **2023,** 297, 127370.
